# Physical activity and cohabitation status moderate the link between diabetes mellitus and cognitive performance in a community-dwelling elderly population in Germany

**DOI:** 10.1371/journal.pone.0187119

**Published:** 2017-10-26

**Authors:** Anne Fink, Nikolaus Buchmann, Christina Tegeler, Elisabeth Steinhagen-Thiessen, Ilja Demuth, Gabriele Doblhammer

**Affiliations:** 1 German Center for Neurodegenerative Diseases, Bonn, Germany; 2 Rostock Center for the Study of Demographic Change, Rostock, Germany; 3 Lipid Clinic at the Interdisciplinary Metabolism Center, Charité–Universitätsmedizin Berlin, corporate member of Freie Universität Berlin, Humboldt-Universität zu Berlin, and Berlin Institute of Health, Berlin, Germany; 4 Klinik und Poliklinik für Innere Medizin B, University of Greifswald, Greifswald, Germany; 5 Bereich Geriatrie der Universitätsmedizin Greifswald, University of Greifswald, Greifswald, Germany; 6 Institute for Sociology and Demography, University of Rostock, Rostock, Germany; Ehime University Graduate School of Medicine, JAPAN

## Abstract

**Aims/Hypothesis:**

The increasing number of people with dementia and cognitive impairments makes it essential to detect and prevent modifiable risk factors of dementia. This study focuses on type 2 diabetes mellitus, especially on undiagnosed cases and their increased risk of cognitive impairment. Furthermore, the potential of physical activity and social integration to moderate the relation between diabetes and cognitive impairment is assessed.

**Methods:**

We used cross-sectional data from 1299 participants of the Berlin Aging Study II (BASE-II) aged between 60 to 84 years and performed logistic regression models to analyze the association of diabetes status, physical activity, and cohabitation status with poor cognitive performance. Cognitive performance was measured with the Consortium to Establish a Registry for Alzheimer's Disease (CERAD)-Plus test battery.

**Results:**

Undiagnosed diabetes (odds ratio (OR) = 2.12, p = 0.031), physical inactivity (OR = 1.43, p = 0.008) and non-cohabiting (OR = 1.58, p = 0.002) were associated with an increased likelihood of poor cognitive performance. The highest odds were observed in participants who suffered from undiagnosed or insulin-dependent diabetes and, in addition, were inactive (undiagnosed diabetes: OR = 3.44, p = 0.003; insulin-dependent diabetes: OR = 6.19, p = 0.019) or lived alone (undiagnosed diabetes: OR = 4.46, p = 0.006; insulin-dependent diabetes: OR = 6.46 p = 0.052). Physical activity and cohabiting appeared to be beneficial.

**Conclusions/Interpretation:**

Physical activity and cohabitation status moderate the link between diabetes mellitus and cognitive performance. Special attention should be paid to undiagnosed and insulin-dependent diabetes cases, which have a particularly high risk of poor cognitive performance.

## Introduction

Cognitive impairments and dementia are among the leading risk factors for disability and death [1–3]. The increase of the number of people living to high ages, when cognitive deficits and related diseases are most prominent, will inevitably lead to an increase in the number of people who have cognitive impairments and dementia. Under the assumption of constant age-specific prevalence, the number of persons with dementia will multiply over the next decades [4]. However, a reduction of age-specific prevalence may substantially diminish the number of affected people [5]. In a meta-analysis Norton and colleagues [6] showed that about one third of all Alzheimer disease (AD) cases are attributable to modifiable risk factors, and that a considerable number of dementia cases could be prevented in the future. As shown by longitudinal analyses, the presence of type 2 diabetes is associated with cognitive dysfunction [7–9] which may be a precursor of mild cognitive impairment (MCI) and dementia. Diabetics have significant lower scores in cognitive test batteries [10] and moderate performance decrements compared to non-diabetics [7, 8]. Especially, the cognitive domains memory, executive function and psychomotor speed have been found to be negatively affected by type 2 diabetes mellitus [9]. Even among non-diabetics, higher glucose levels are associated with an increased dementia risk [11]. There is an increased risk of conversion to dementia in diabetes patients, with a higher risk of conversion to vascular dementia (VaD) than to AD [12, 13]. Diabetics with MCI are more likely to develop dementia or AD than are non-diabetics with MCI [14]. However, effective glycemic control is correlated with a reduced risk of cognitive dysfunction and dementia [15, 16]. The underlying mechanisms between diabetes and dementia do not seem to be monocausal. Pathways via atherosclerosis, microvascular diseases, and the impact of glucose toxicity and insulin resistance of diabetics are suspected of leading to brain pathologies which cause vascular dementia, AD, or mixed forms [17]. Studies reported that the prevalence of diabetes has been increasing over the last decades and a substantial number of people live with undiagnosed diabetes [18–20]. The resulting lack of glycemic control means that undiagnosed diabetes increases the risk of all dementias, AD, and VaD [16].

Another important aspect of a diabetes related life style factor is regular physical activity. Numerous studies have proven the beneficial effects of physical activity on cognition [21–23]. A meta-analysis reported a 1.82-fold increased risk of AD if people were physically inactive [6]. Risks of cognitive impairment or decline were significantly reduced for persons with high levels of physical activity [24].

In addition to physical activity, the cohabitation status and levels of social integration also affect the development of cognitive decline [22, 25]. People who cohabited and/or were married had the lowest risks for MCI, AD, and cognitive decline [26, 27].

In the present study we analyze whether the presence of diabetes mellitus, physical activity, and cohabitation status are correlated with cognitive performance for members of a community-dwelling elderly population. We differentiate between non-diabetics, diabetics treated with oral anti-diabetic medications (ADM), diabetics treated with insulin, untreated diabetics, and undiagnosed diabetics. We hypothesize that diabetics with effective glycemic control have comparable risks of poor cognitive performance when compared to non-diabetics, whereas insulin-dependent diabetics show increased risks of cognitive impairment as they are probably in a later and more severe stage of the disease [28]. In a longitudinal analysis of health claims data, insulin-dependent diabetics had a 60% increased risk of dementia [15]. To identify persons at high risk, we focus on undiagnosed diabetes, which is assumed to pose the highest risks for cognitive impairments as those affected do not know about their disease and do not have good glycemic control. Furthermore, we assume that regular physical activity and cohabiting may moderate the link between diabetes and cognitive performance.

## Materials and methods

### Data

The Berlin Aging Study II (BASE-II) is an ongoing joint project of various disciplines involving several institutions. The population-based sample of community-dwelling participants, living in the greater metropolitan area of Berlin, Germany, covers numerous ageing-relevant variables [29]. The sample consists of 600 younger individuals ages 20 to 35 and 1600 older individuals ages 60 to 84 (for a detailed description of the study see Bertram and colleagues and Gerstorf and colleagues [29, 30]). All participants gave written informed consent to participation and the Ethics Committee of the Charité-Universitätsmedizin Berlin approved this study (approval number EA2/029/09).

#### Analytical sample

For our analyses we used data of the older subsample and included only those individuals with complete information on diabetes status, physical activity, cohabitation status, and the neuropsychological test battery, resulting in an analytical sample of 1299 participants.

#### Measurement of cognitive performance

To measure cognitive performance we used the German version of the neuropsychological test battery CERAD-Plus (Consortium to Establish a Registry for Alzheimer's Disease) [31–33]. The complete test battery was administered to all 1299 participants studied here. The following tests were used to evaluate the cognitive performance of the subjects: Word list learning, word list recall, constructional praxis, recall of constructional praxis, verbal fluency, phonemic fluency, Trail Making Test A and Trail Making Test B. We applied the following standardization to all scales

$${value}_{stand}=\frac{\left(value-{value}_{min}\right)}{\left({value}_{max}-value\right)}$$

and reversed the order of the values of Trail Making Tests A and B so that higher values correspond to better test performances. Creating an index that reflects the overall cognitive performance of the participants, we summed up the standardized values of all tests for each person. Previous studies showed that a total CERAD score may differentiate between normal controls and MCI subjects better than the Mini Mental Status Examination (MMSE) because of ceiling effects [34, 35]. In our sample, only two of the 1299 participants had a MMSE score of less than 24 points, which is an established cut-off point for a conspicuously impaired cognitive function. We dichotomized the total CERAD score index and labeled the lowest 25 percent of the distribution as ‘‘poor performance”.

#### Definition of diabetes mellitus

Study participants were defined as diabetic if one of the following criteria was fulfilled: (1) Subjects listed a diabetes diagnosis in the questionnaire; (2) Intake of an oral anti-diabetic drug or insulin; (3) Glycated haemoglobin (HbA1c) levels over 6.5%; (4) Fasting plasma glucose (FPG) over 126 mg/dL; (5) 2-hour glucose level over 200 mg/dL [36]. The 2-hour test was only administered to people who did not state a diagnosis of diabetes in the questionnaire. Because we know whether anti-diabetic medications were prescribed to participants, we divided them into five categories: Non-diabetics, diagnosed diabetics treated with oral anti-diabetic medications (ADM), diagnosed diabetics treated with insulin, diagnosed diabetics without any medical treatment, and persons with undiagnosed diabetes as of study participation.

#### Physical activity and cohabitation status

Physical activity was assessed with the Rapid Assessment of Physical Activity (RAPA) questionnaire [37, 38]. We defined subjects who stated “I do 30 minutes or more a day of moderate physical activities, 5 or more days a week” or “I do 20 minutes or more a day of vigorous physical activities, 3 or more days a week” as active.

The cohabitation status was assessed with the question “How do you live?”. We distinguished between people who lived together with a partner or a relative (labeled as “not alone”) and subjects living alone.

#### Covariates

The following covariates were entered into the statistical analyses: Sex; age in years as metric variable (ranging from 60 to 84); education (high = 12 or more years of education and/or having a higher education entrance qualification vs. low = less than 12 years of education); body mass index (less than 30 kg/m^2^ vs. 30 kg/m^2^ or more) [39]; current smoking (smoker vs. non-smoker); self-reported hypertension (yes vs. no), self-reported history of stroke (yes vs. no), self-reported cardiovascular diseases (none vs. at least one cardiovascular disease: coronary heart disease, cardiac insufficiency, peripheral arterial disease, impaired cerebral blood flow, or myocardial infarction); depression (yes vs. no: self-reported or 16 or more points on the CESD-scale [40]); and self-reported dyslipidemia (yes vs. no).

### Statistical analyses

We compared the characteristics of the participants by their diabetes status and used one-way ANOVA for continuous variables and χ^2^ tests for categorical variables. We performed univariate logistic regression models with all used covariates and three multivariate logistic regression models for calculating the odds ratios (OR) of poor cognitive performance. With the exception of age, all independent variables were included as dummy variables. We extended our models by interaction terms in order to test for moderator effects of physical activity and cohabitation status with diabetes mellitus. All analyses were performed using STATA 12.1.

## Results

### Descriptive results

**Fig 1:**
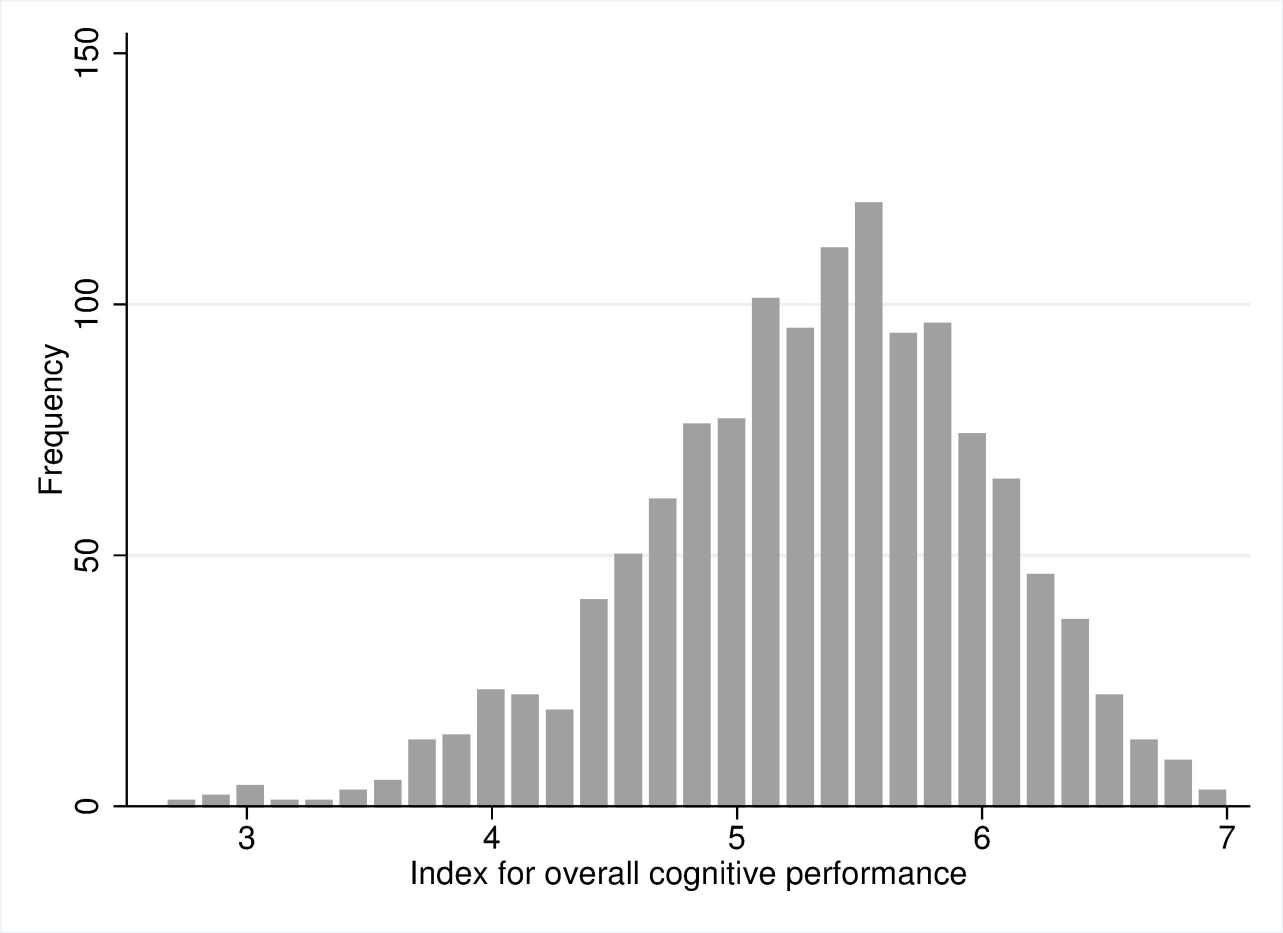
Distribution of the summary index of overall cognitive performance. Source: BASE-II.

Fig 1 displays the distribution of the CERAD score index, which is approximately normally distributed. The index ranges from 2.6, indicating a low overall cognitive performance, to 7, indicating a high overall cognitive performance. In our sample, 325 persons had a poor cognitive performance (lowest 25%), 974 were defined to have a good cognitive performance. Table 1 portrays the distribution of all independent variables by the cognitive performance of the participants. Of the 659 men and 640 women, 12.7% were diabetics and about half of these (6%) were not being treated with any ADM. 3.1% of the participants did not know about their condition prior to the study participation. 48.3% had an active life style and 61.5% lived together with the partner or a relative. A distinction by diabetes status revealed that the five diabetes groups differ significantly regarding their HbA1c, FPG, and 2-hour glucose levels (Table 2). Post hoc tests using Bonferroni revealed significant mean differences in HbA1c levels between all treatment groups with the exception of untreated and undiagnosed diabetics. Regarding the FPG level, mean differences did not reach statistical significance between untreated diabetics, undiagnosed diabetics and diabetics treated with oral ADM (S1 Table–S3 Table). Insulin-dependent diabetics had highest average HbA1c and FPG levels. The 2-hour glucose level was significantly higher for undiagnosed diabetics compared to non-diabetics. Case numbers for these three parameters differ, as not all values were available for all participants. Untreated and undiagnosed diabetics were most often physically inactive. The five diabetes groups did not differ significantly regarding cohabitation status.

**Table 1:** Characteristics of the study population of BASE-II, N = 1299. Source: BASE-II.

		Poor cognitive performance		Good cognitive performance		Total	
	
Variable		n	%	n	%	n	%
Diabetes mellitus							
	Non-diabetics	266	81.9	868	89.1	1,134	87.3
	Treated diabetics, oral ADM	22	6.8	46	4.7	68	5.2
	Treated diabetics, insulin	7	2.2	12	1.2	19	1.5
	Untreated diabetics	13	4.0	25	2.6	38	2.9
	Undiagnosed diabetics	17	5.2	23	2.4	40	3.1
Physical activity							
	Active	130	40.0	497	51.0	627	48.3
	Inactive	195	60.0	477	49.0	672	51.7
Cohabitation status							
	Not alone	183	56.3	616	63.2	799	61.5
	Alone	142	43.7	358	36.8	500	38.5
Sex							
	Male	187	57.5	472	48.5	659	50.7
	Female	138	42.5	502	51.5	640	49.3
							
Mean age in years (sd)		68.98 (0.21)		67.75 (0.11)		68.06 (0.10)	
							
MMSE							
	<24	2	0.6	2	0.2	4	0.3
	24–30	323	99.4	970	99.6	1,293	99.5
	Missing	0	0.0	2	0.2	2	0.2
Education							
	Low	216	66.5	471	48.4	687	52.9
	High	109	33.5	503	51.6	612	47.1
Hypertension							
	No	159	48.9	545	56.0	704	54.2
	Yes	166	51.1	429	44.1	595	45.8
Stroke							
	No	317	97.5	954	98.0	1271	97.8
	Yes	8	2.5	20	2.1	28	2.2
Cardiovascular diseases							
	None	293	90.2	891	91.5	1184	91.2
	At least one	32	9.9	83	8.5	115	8.9
Depression							
	No	190	58.5	602	61.8	792	61.0
	Yes	128	39.4	367	37.7	495	38.1
	Missing	7	2.2	5	0.5	12	0.9
Dyslipidemia							
	No	207	63.7	608	62.4	815	62.7
	Yes	118	36.3	366	37.6	484	37.3
Body mass index							
	<30 kg/m^2^	258	79.4	809	83.1	1,067	82.1
	> = 30 kg/m^2^	67	20.6	165	16.9	232	17.9
Current smoking							
	No	290	89.2	887	91.1	1,177	90.6
	Yes	35	10.8	87	8.9	122	9.4

sd = standard deviation

ADM = anti-diabetic medications

MMSE = Mini Mental Status Examination

**Table 2:** Characteristics of the study population by diabetes status, N = 1299. Source: BASE-II.

Characteristics	N	Non-diabetics	Treated diabetics, oral ADM	Treated diabetics, insulin	Untreated diabetics	Un-diagnosed diabetics	p-value
HbA1c (mean; sd)	1261	5.5; 0.4	6.6; 0.8	7.3; 1.1	6.2; 0.8	6.3; 0.6	<0.001
FPG in mg/dL (mean; sd)	1278	91.3; 9.4	129.6; 31.7	151.2; 61.4	121.2; 27.0	123.2; 26.2	<0.001
2-hour glucose in mg/dL (mean; sd)	1115	103.7; 28.3	—	—	—	201.8; 66.2	<0.001
Physically inactive (%)	1299	50.4	55.9	42.1	71.1	70.0	0.011
Living alone (%)	1299	39.3	30.9	26.3	31.6	40.0	0.407
Men (%)	1299	49.0	67.7	79.0	55.3	52.5	0.004
Age in years (mean; sd)	1299	68.1; 3.6	67.6; 3.5	68.1; 3.3	68.2; 4.1	68.1; 4.1	0.862
High education (%)	1299	48.0	35.3	52.6	42.1	45.0	0.305
Hypertension (%)	1299	42.0	73.5	79.0	84.2	55.0	<0.001
Stroke (%)	1299	2.1	4.4	5.3	0.0	0.0	0.375
Cardiovascular diseases (%)	1299	8.2	10.3	42.1	13.2	5.0	<0.001
Depression (%)	1299	37.7	45.6	47.4	34.2	37.5	0.701
Dyslipidemia (%)	1299	34.8	63.2	57.9	52.6	37.5	<0.001
BMI≥30 kg/m^2^ (%)	1299	14.7	36.8	52.6	39.5	37.5	<0.001
Current smoking (%)	1299	9.2	14.7	0.0	2.6	17.5	0.051

sd = standard deviation

ADM = anti-diabetic medications

HbA1c = glycated haemoglobin

BMI = body mass index

FPG = fasting plasma glucose

### Model results

Univariate logistic regression analysis revealed that diabetics compared to non-diabetics had a statistically increased odds ratio (OR) of 1.82 (p = 0.001) of poor cognitive performance (Table 3). Table 4 presents our main results in the form of OR of poor cognitive performance dependent on the diabetes status. Model 1 is adjusted for sex, age, and education only. All other models are adjusted for sex, age, education, hypertension, stroke, cardiovascular diseases, depression, dyslipidemia, body mass index, and current smoking. Differentiation by the treatment of the diabetics revealed that undiagnosed diabetes is associated with a particularly high odds ratio of poor cognitive performance (Model 3: OR = 2.12, p = 0.031). Persons receiving oral ADM and untreated diabetics also showed elevated odds ratios, but estimates did not reach statistical significance. Although not statistically significant, the odds ratio for insulin-dependent diabetics was quite high (OR = 1.95, p = 0.193). The results are stable and do not change much when controlling for covariates. Being inactive was significantly correlated with poor overall cognitive performance (OR = 1.43, p = 0.008). People living alone had an increased odds ratio of 1.58 (p = 0.002).

**Table 3:** Univariate odds ratios of poor cognitive performance, N = 1,299. Source BASE-II.

Variable		OR	p-value
Diabetes mellitus			
	Non-diabetics (RG)	1.00	
	Diabetics	1.82	0.001
Diabetes mellitus			
	Non-diabetics (RG)	1.00	
	Treated diabetics, oral ADM	1.56	0.097
	Treated diabetics, insulin	1.90	0.181
	Untreated diabetics	1.70	0.130
	Undiagnosed diabetics	2.41	0.007
Physical activity			
	Active (RG)	1.00	
	Inactive	1.56	0.001
Cohabitation status			
	Not alone (RG)	1.00	
	Alone	1.34	0.026
Sex			
	Men (RG)	1.00	
	Women	0.69	0.005
			
Age in years		1.10	<0.001
			
Education			
	Low (RG)	1.00	
	High	0.47	<0.001
Hypertension			
	No (RG)	1.00	
	Yes	1.33	0.028
Stroke			
	No (RG)	1.00	
	Yes	1.20	0.661
Cardiovascular diseases			
	None (RG)	1.00	
	At least one	1.17	0.467
Depression			
	No (RG)	1.00	
	Yes	1.11	0.450
	Missing	4.44	0.012
Dyslipidemia			
	No (RG)	1.00	
	Yes	0.95	0.682
Body mass index			
	<30 kg/m^2^ (RG)	1.00	
	> = 30 kg/m^2^	1.27	0.135
Current smoking			
	No (RG)	1.00	
	Yes	1.23	0.326

RG = Reference group

ADM = anti-diabetic medications

OR = odds ratio

**Table 4:** Odds Ratios of poor cognitive performance, N = 1299. Source: BASE-II.

		Model 1 *		Model 2 **		Model 3**	
Variable		OR	p-value	OR	p-value	OR	p-value
Diabetes mellitus							
	Non-diabetics (RG)	1.00		1.00		1.00	
	Treated diabetics, oral ADM	1.42	0.210	1.34	0.314	1.34	0.317
	Treated diabetics, insulin	1.88	0.202	1.80	0.247	1.95	0.193
	Untreated diabetics	1.61	0.189	1.51	0.270	1.48	0.299
	Undiagnosed diabetics	2.43	0.009	2.22	0.020	2.12	0.031
Physical activity							
	Active (RG)					1.00	
	Inactive					1.43	0.008
Cohabitation status							
	Not alone (RG)					1.00	
	Alone					1.58	0.002
Hosmer-Lemeshow-Chi² (df = 8)		5.13		12.77		4.25	
p-value(Hosmer-Lemeshow-Chi²)		0.743		0.120		0.834	

*Adjusted for sex, age, education

**Adjusted for sex, age, education, hypertension, stroke, cardiovascular diseases, depression, dyslipidemia, body mass index, current smoking

RG = Reference group

ADM = anti-diabetic medications

OR = odds ratio

df = degrees of freedom

### Model results with interaction terms

**Fig 2:**
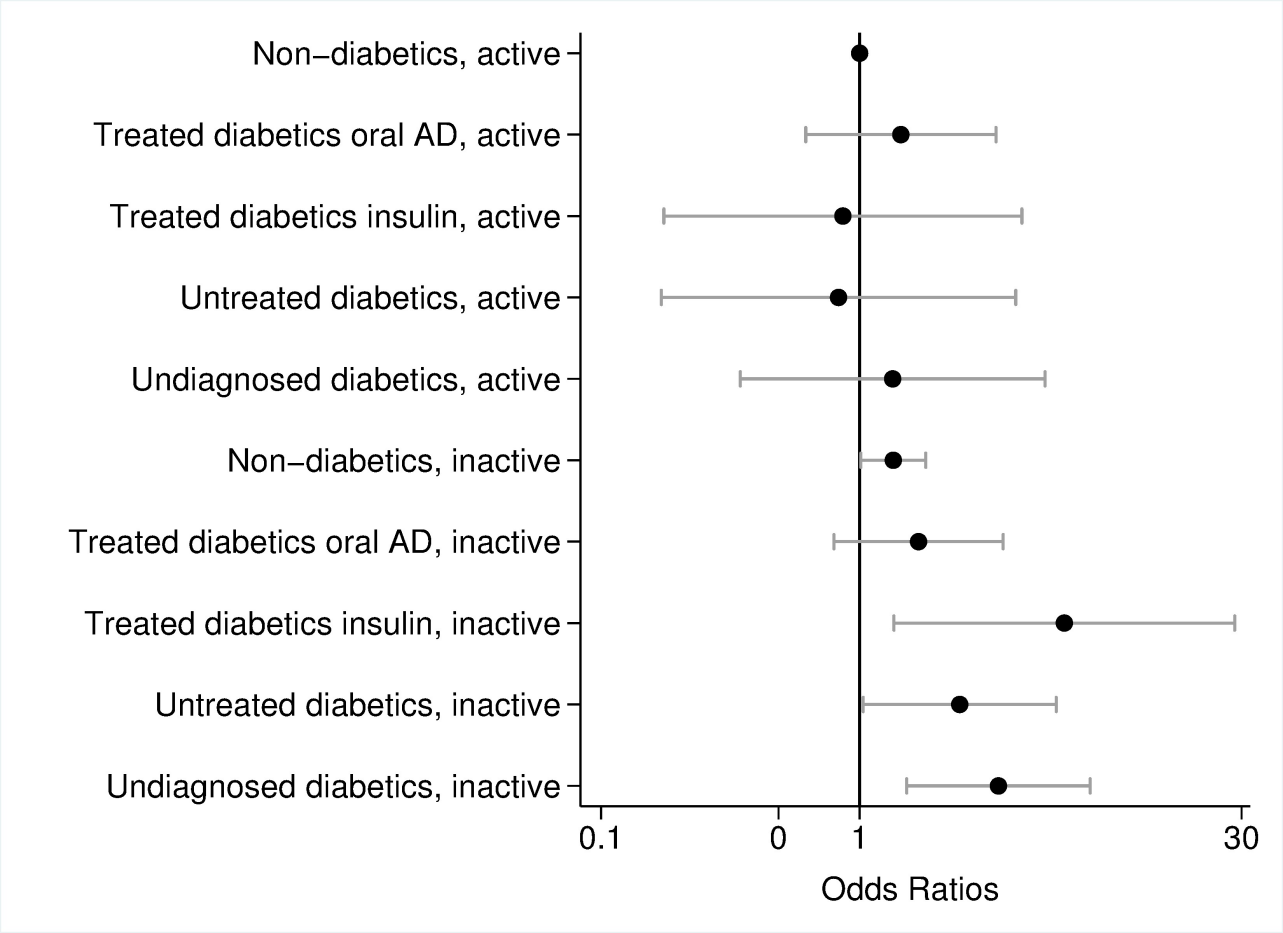
Odds ratios and 95% confidence interval of poor cognitive performance, interaction effects of diabetes mellitus and physical activity adjusted for sex, age, education, hypertension, stroke, cardiovascular diseases, depression, dyslipidemia, body mass index, current smoking; logarithmic scale. Error bars represent 95% confidence interval. Source: BASE-II.

**Fig 3:**
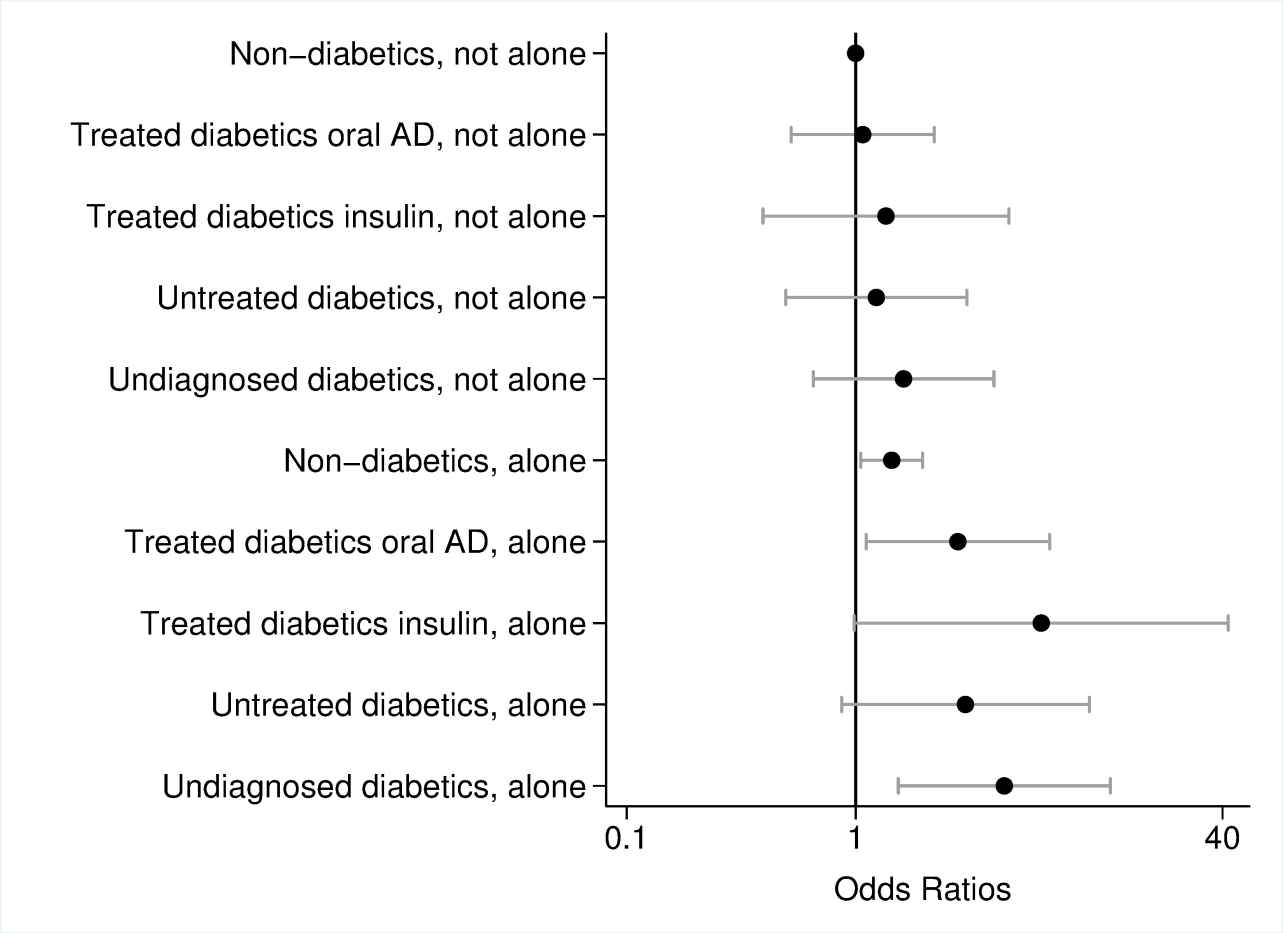
Odds ratios and 95% confidence interval of poor cognitive performance, interaction effects of diabetes mellitus and cohabitation status adjusted for sex, age, education, hypertension, stroke, cardiovascular diseases, depression, dyslipidemia, body mass index, current smoking; logarithmic scale. Error bars represent 95% confidence interval. Source: BASE-II.

Models with interaction terms revealed that the likelihood of poor overall cognitive performance was particularly high if people suffered from undiagnosed diabetes (OR = 3.44, p = 0.003) or were treated with insulin (OR = 6.19, p = 0.019) and were also inactive (Fig 2). Also inactive non-diabetics and inactive untreated diabetics had increased odds ratios (non-diabetics: OR = 1.35, p = 0.042; untreated diabetics: OR = 2.44, p = 0.042) compared to active non-diabetics. However, physically active persons did not have an increased likelihood of poor overall cognitive performance independent of their diabetes status. Fig 3 presents model results of the interaction between diabetes and cohabitation. Living alone increased the risk of poor overall cognitive performance for non-diabetics (OR = 1.44, p = 0.022) as well as for diabetics treated with oral ADM (OR = 2.79, p = 0.029) and undiagnosed diabetics (OR = 4.46, p = 0.006), whereas effects were highest for insulin-dependent diabetics who lived alone (OR = 6.46, p = 0.052). Diabetic people who cohabitate had an odds ratio of poor cognitive performance comparable to non-diabetics.

## Discussion

In the current study undiagnosed diabetics without effective glycemic control were shown to have the highest risks of poor cognitive performance, which is in line with a previous study by Xu and colleagues [16]. A considerable proportion, almost one quarter, of all diabetes cases did not know about their disease prior to participation in this study. Insulin-dependent diabetics also showed highly elevated odds ratios of poor cognitive performance, but most likely due to the small case numbers these estimates did not reach statistical significance. Intake of insulin seems to be an indicator for the severity of the disease, with insulin-dependent diabetics at a more severe stage [28]. Both undiagnosed and insulin-dependent diabetics had the highest HbA1c, FPG, or 2-hour glucose levels, which indicates insufficient or no glycemic control. In contrast, diabetics treated with oral ADM and untreated diabetics had comparable risks of poor cognitive performance compared to non-diabetics. These persons are probably still in a mild stage of the disease, and effective glycemic control seems to be achieved either by oral ADM or by nutrition. Previous studies have shown that treatment with oral ADM is beneficial for cognition and may attenuate the harmful effects of diabetes in patients [15, 16]. A meta-analysis revealed that diets of low-carbohydrates, a low-glycemic index, Mediterranean, and high in protein are effective in glycemic control [41] and may therefore reduce the risk of cognitive impairment in early stages of the disease as compared to diabetics with insufficient or no glycemic control.

Along with the status of diabetes, life style variables had an influence on the likelihood of poor cognitive performance. People who were inactive and living alone have significantly elevated risks, which is in line previous studies [21–26]. Fratiglioni and colleagues [22] proposed three hypotheses about how social integration and physical activity might offer protection from cognitive impairment and dementia. First, the cognitive-reserve hypothesis postulates that physical activity and social interactions enhance the plasticity of the brain and compensatory functions, therefore perhaps preventing cognitive decline. Second, the vascular hypothesis describes the beneficial effect of physical activity and social integration on the pathogenesis of cardiovascular diseases, which are in turn risk factors for cognitive impairment and dementia. Third, the stress hypothesis assumes that physically active and socially integrated people show lower levels of stress and can better cope with stress. A failure of stress adaption does indeed matter in the development of cognitive decline and dementia [22].

The combination of undiagnosed or insulin-dependent diabetes and inactivity or non-cohabitation was particularly deleterious. However, if participants were regularly physically active or lived together with a partner or relative, the risks of poor cognitive performance did not differ from those of active or cohabiting non-diabetics. This result was independent of the treatment status of the diabetics. Still, the enhanced risk due to undiagnosed or insulin-dependent diabetes could partly be compensated for by physical activity and social integration. Both factors most likely counteract the harmful effects of diabetes and insufficient glycemic control. This finding is in line with a recent a study indicating that moderate-to-high intensity aerobic exercise may have a disease-modifying effect in terms of reduced levels of tau proteins in cerebrospinal fluid, increases in blood flow in the brain, and improvements of executive functions in elderly people with MCI and prediabetes [42].

The present study does have some limitations. First, the cross-sectional design does not allow us to draw causal conclusions. There is always the possibility of reverse causation, meaning that cognitive impairment is the cause of an unhealthy life style which leads to diabetes or inactivity. Second, social integration was assessed in conjunction with the cohabitation status. Living alone does not necessarily mean that people are not socially integrated. Nevertheless, cohabiting is one of the main components of social interaction [43] and we were able to demonstrate the positive effect of living with a partner or relative. Third, we used a relatively strict definition of physical activity. However, a sensitivity analysis with a wider definition that also classified people as active who report doing”moderate physical activities every week, but less than 30 minutes a day or 5 days a week” or “vigorous physical activities every week, but less than 20 minutes a day or 3 days a week” showed similar effects. Fourth, in a life-course perspective, untreated diabetics may receive drug treatment sooner or later and would then belong to the group of treated diabetics. There might also be social selection forces, such as education or income, which would increase the likelihood of receiving a diabetes diagnosis and thus interplay with the positive effect of the treatment and compliance of the patients. Especially, the group of undiagnosed diabetics may have a generally lower health literacy and the lack of their diabetes diagnosis may potentially be associated with also undiagnosed cardiovascular diseases or dyslipidemia which were self-reported. This should be in mind when interpreting estimates of cognitive performance of this group.

The strength of this study is the large number of cases with available information on their cognitive status measured with a total score of the CERAD-Plus test battery, which is superior to the MMSE in detecting MCI [34, 35]. The advantage of a summary score is that every subtest carries the same weight. The score gives a full picture of a participant’s cognitive performance. On the other hand, the total score does not allow an evaluation of the performance in special cognitive domains. The in-depth medical anamnesis and the numerous laboratory values allow us to reliably identify undiagnosed diabetes cases, which in turn enabled us to differentiate between diagnosed diabetics receiving no drug treatment and undiagnosed diabetics who also were untreated. A comparison of demographic characteristics with the German general population revealed that the BASE-II sample is relatively healthy and well educated [29]. The effects of diabetes, physical activity, and cohabitation status on cognitive performance are therefore potentially underestimated.

Several studies have indicated a decreasing trend of dementia prevalence and incidence [44–50], primarily due to higher educational levels and a reduction of vascular risk factors, especially stroke [51]. Nevertheless, it is questionable whether these trends will continue as the rising diabetes prevalence could counteract successes in the prevention of other cardiovascular diseases and thus the observed trends. Consequently, it is essential to detect and treat type 2 diabetes mellitus as early as possible, not only to prevent cognitive decline and subsequent dementia but also other complications which might be caused by diabetes, for example retinopathy, nephropathy, or polyneuropathy [20]. In addition, physical activity and social integration play an important role in helping to overcome the problem of an increasing prevalence of diabetes. An early screening for diabetes, especially in patients with overweight or familial history of diabetes, may be useful. Beyond screening and providing comprehensive information to the population targeted life style intervention strategies for diabetic patients are valuable [52]. Interventions such as an increased physical activity, dietary education and counseling for treatment adherence showed beneficial effects on risk factors such as high BMI and HbA1c values in diabetic patients [53]. In the case of frequent hyperglycemic phases, diabetic patients could receive targeted trainings in outpatient clinics. If such courses were held in small groups, this would also promote social interaction.

A healthy life style and goal-directed detection and treatment of diabetes might contribute to the continuation of decreasing incidence and prevalence of dementia. Further research is needed to analyze to what extent these factors influence the development of cognitive impairments and dementia.

## Supporting information

S1 Table Posthoc mean comparisons of HbA1c by diabetes status using Bonferroni, p-values shown. Source: BASE-II.
(DOCX)

S2 Table Posthoc mean comparisons of fasting plasma glucose by diabetes status using Bonferroni, p-values shown. Source: BASE-II.
(DOCX)

S3 Table Posthoc mean comparisons of age by diabetes status using Bonferroni, p-values shown. Source: BASE-II.
(DOCX)
