# Correction: Physical activity and cohabitation status moderate the link between diabetes mellitus and cognitive performance in a community-dwelling elderly population in Germany

**DOI:** 10.1371/journal.pone.0349833

**Published:** 2026-05-21

**Authors:** Anne Fink, Nikolaus Buchmann, Christina Tegeler, Elisabeth Steinhagen-Thiessen, Ilja Demuth, Gabriele Doblhammer

After this article [1] was published, it came to light that the study used an unauthorized version of the German MMSE questionnaire [2]. The study [1] used the version of the German MMSE from the Swiss Memory Clinic [2], which includes the MMSE as part of the CERAD-Plus test battery (which was also used in [1]). This issue was resolved in post-publication discussions where evidence was provided that a license to use the unauthorized version of the German MMSE in [1] was obtained retrospectively from Psychological Assessment Resources (PAR), who has the rights to publish, license, and manage intellectual property rights for the MMSE [3].

The Acknowledgements section of [1] is corrected to: Thanks to all employees and participants of BASE-II who supported this study by their contributions, discussions, and cooperation. An unauthorized version of the German MMSE was used by the study team without permission and has been rectified with PAR. The MMSE is a copyrighted instrument and may not be used or reproduced in whole or in part, in any form or language, or by any means without written permission of PAR (www.parinc.com).

## References

[1] Fink A, Buchmann N, Tegeler C, Steinhagen-Thiessen E, Demuth I, Doblhammer G. Physical activity and cohabitation status moderate the link between diabetes mellitus and cognitive performance in a community-dwelling elderly population in Germany. PLoS One. 2017;12(10):e0187119. doi: 10.1371/journal.pone.0187119 29073237 PMC5658168

[2] Memory Clinic. Die Neuropsychologische Testbatterie CERAD-Plus. [Accessed April 2026]. Available from: https://www.memoryclinic.ch/de/main-navigation/neuropsychologen/cerad-plus/

[3] Folstein MF, Folstein SE, McHugh PR. “Mini-mental state”. A practical method for grading the cognitive state of patients for the clinician. J Psychiatr Res. 1975;12(3):189–98. doi: 10.1016/0022-3956(75)90026-6 1202204

