# Supplementary material for: Physical activity and cohabitation status moderate the link between diabetes mellitus and cognitive performance in a community-dwelling elderly population in Germany
Source: PLoS One. 2017 Oct 26;12(10):e0187119. doi: 10.1371/journal.pone.0187119 (PMC5658168; doi:10.1371/journal.pone.0187119)
Supplement: S2 Table — Source: BASE-II. (DOCX) [file pone.0187119.s002.docx]

|  | Non-diabetics | Treated diabetics, oral ADM | Treated diabetics, insulin | Untreated diabetics |
| --- | --- | --- | --- | --- |
| Treated diabetics, oral ADM | <0.001 |  |  |  |
| Treated diabetics, insulin | <0.001 | <0.001 |  |  |
| Untreated diabetics | <0.001 | 0.060 | <0.001 |  |
| Un-diagnosed diabetics | <0.001 | 0.311 | <0.001 | 1.000 |

ADM=anti-diabetic medications
